# Targeting AGEs Signaling Ameliorates Central Nervous System Diabetic Complications in Rats

**DOI:** 10.1155/2015/346259

**Published:** 2015-09-29

**Authors:** Mohamed Naguib Zakaria, Hany M. El-Bassossy, Waleed Barakat

**Affiliations:** ^1^Department of Pharmacology and Toxicology, Faculty of Pharmacy, Zagazig University, Zagazig 44519, Egypt; ^2^Department of Pharmacology, Faculty of Pharmacy, King Abdulaziz University, Jeddah 80200, Saudi Arabia; ^3^Department of Pharmacology, Faculty of Pharmacy, University of Tabuk, Tabuk 71491, Saudi Arabia

## Abstract

Diabetes is a chronic endocrine disorder associated with several complications as hypertension, advanced brain aging, and cognitive decline. Accumulation of advanced glycation end products (AGEs) is an important mechanism that mediates diabetic complications. Upon binding to their receptor (RAGE), AGEs mediate oxidative stress and/or cause cross-linking with proteins in blood vessels and brain tissues. The current investigation was designed to investigate the effect of agents that decrease AGEs signaling, perindopril which increases soluble RAGE (sRAGE) and alagebrium which cleaves AGEs cross-links, compared to the standard antidiabetic drug, gliclazide, on the vascular and *central nervous system* (CNS) complications in STZ-induced (50 mg/kg, IP) diabetes in rats. Perindopril ameliorated the elevation in blood pressure seen in diabetic animals. In addition, both perindopril and alagebrium significantly inhibited memory decline (performance in the Y-maze), neuronal degeneration (Fluoro-Jade staining), AGEs accumulation in serum and brain, and brain oxidative stress (level of reduced glutathione and activities of catalase and malondialdehyde). These results suggest that blockade of AGEs signaling after diabetes induction in rats is effective in reducing diabetic CNS complications.

## 1. Introduction

Diabetes mellitus is an endocrine disorder resulting from inadequate insulin release or insulin insensitivity [1]. The prevalence of diabetes worldwide was estimated to be 2.8% in 2000 and 4.4% in 2030 [2]. The prevalence of diabetes in Egypt was 3.9% in 2000 and is expected to rise to 6.8% by the year 2030 which would make Egypt one of the highest 10 countries with diabetes in 2030 [2].

Diabetes often results in microvascular and macrovascular complications such as retinopathy, peripheral neuropathy, stroke, and coronary heart disease [1]. Hypertension is very frequently associated with diabetic subjects, irrespective of whether they are type 1 or type 2 [3]. Diabetes induces advanced brain aging and may ultimately result in deficits in cognitive performance [4] and increased risk of developing clinical manifestations of Alzheimer's disease [5].

Although several drugs are available to control elevated blood glucose level in diabetic patients, many diabetic patients still suffer from diabetic complications and so new treatment strategies are required to manipulate these chronic widely spreading complications.

Poor glycemic control increases the accumulation of advanced glycation end products (AGEs) [6] and oxidative stress, which may lead to cellular and molecular damage [7] that contributes to diabetes-induced brain aging [8]. AGEs have been associated with increased oxidative stress [9] and inhibition of reactive oxygen species (ROS) was shown to interfere with the formation of AGEs [10]. AGEs activate the receptor for advanced glycation end products (RAGE) [11] which is implicated in the pathogenesis and progression of chronic diseases such as diabetes and immune/inflammatory disorders [12]. In addition, RAGE expression was increased in human diabetic kidney [13].

The AGE/RAGE interaction promotes reactive oxygen species (ROS) production [14] and activates protein kinase C (PKC) and nuclear factor-kappa B (NF-*κ*B) [15]. In addition, ROS themselves may fuel further generation of AGEs [16]. Soluble RAGE (sRAGE) is the extracellular ligand-binding domain of RAGE that binds ligands and blocks their interaction with, and activation of, cell surface receptors [11]. Chronic administration of sRAGE protects against macro- and microvascular complications in the great vessels, heart, kidney, retina, and peripheral nerve [12].

Among other actions of AGEs are effects on extracellular matrix proteins and basement membrane components and formation of protein cross-links which can cause or facilitate vascular complications [17, 18]. The AGE cross-link breaker alagebrium (ALT-711) has been shown to cleave preformed AGE cross-links and reduce tissue levels of AGEs [19] resulting in improved total arterial compliance in aged humans with vascular stiffening [18].

Previous studies have demonstrated a relationship between the renin-angiotensin system (RAS) and the accumulation of AGEs in experimental diabetes [20]. ACE inhibition was shown to reduce the accumulation of serum AGEs in diabetes, possibly via effects on oxidative pathways [20] and by increasing the production and secretion of sRAGE into plasma as shown with perindopril which caused an increase in plasma sRAGE in patients [21].

The present study was designed to investigate the possible effect of agents that decrease AGEs signaling (perindopril which increases sRAGE level or alagebrium which cleaves AGEs-induced protein cross-links) on the impact of diabetes on blood pressure and CNS functions in STZ diabetic rats.

## 2. Material and Methods

### 2.1. Animals

Adult male Wistar rats weighing 170 ± 20 g were obtained from the National Research Institute (Cairo, Egypt). All experimental procedures were approved by the Ethical Committee for Animal Handling at Zagazig University (ECAHZU).

### 2.2. Drugs and Chemicals

STZ was purchased from Sigma-Aldrich (Germany), and perindopril (Coversyl tablets) and gliclazide (Diamicron tablets) were purchased from Servier Egypt Industries, while alagebrium was purchased from Chemos (Germany).

### 2.3. Study Protocol

Diabetes was induced by streptozotocin (STZ, 50 mg/kg, IP) [22] and rats with stable hyperglycemia (300–400 mg/dL) after 8 weeks of STZ injection were randomly distributed among four groups (*n* = 6) and received alagebrium (10 mg/kg) [23, 24], perindopril (4 mg/kg) [25, 26], or gliclazide (10 mg/kg) [27] daily as oral suspension in 0.5% carboxymethyl cellulose (CMC) for another 6 weeks. In addition, 6 nondiabetic rats received similar volume of CMC daily for the same duration and served as control. Eight weeks after diabetes induction was previously shown to be necessary for development of significant vascular complications as shown in our previous studies [28].

### 2.4. Behavior Changes

Behavior changes were assessed in Y-maze as a score: 0, no entrance to target arm; 1, entrance to target arm only and staying in it; 2, entrance to nontarget arm first and then target arm; 3, entrance to target arm first and passing three arms in more than four minutes; 4, entrance to target arm first and passing three arms within four minutes; 5, entrance to target arm first and passing three arms in less than one minute [29, 30].

### 2.5. Blood Glucose and Blood Pressure Measurement

Twelve hours after the last injection, body weight and blood glucose were measured (Glucometer Bionime GM100 Blood Glucose Meter) and blood pressure was recorded (Power Lab 26T, LTS) in a conscious and slightly restrained rat by tail cuff method as previously described [31].

### 2.6. Blood and Tissue Sampling

Blood was collected from the retroorbital plexus under topical ophthalmic anesthetic, centrifuged at 3000 ×g, 4°C for 20 min (Hermle Z326K), and serum was stored at −20°C for later determination of serum AGEs level.

Animals were sacrificed and brain was carefully isolated and frozen at −80°C and 20 *μ*m sections were prepared using cryostat (Slee, Mainz, Germany) and used for the detection of neuronal degeneration by Fluoro-Jade B staining [32].

The whole brain was cut from olfactory bulb to the cerebellum. The distance between sections was 400 *μ*m and the trimmed portion of the brain was homogenated and used for detection of AGEs or oxidative stress biomarkers.

### 2.7. Determination of Neuronal Degeneration by Fluoro-Jade B Staining (FJ-B)

Fluoro-Jade staining was performed using the method described previously [33]. Fluoro-Jade stained slides were visualized under fluorescent microscope (Leica DM500, Leica, Germany). At least ten different fields were photographed from each section and the images were analyzed by ImageJ software.

### 2.8. Detection of AGEs Level in Serum and Brain

Advanced glycation end products (AGEs) level was detected in serum and brain homogenates (extracted with PBS) fluorometrically [34, 35] at excitation wavelength 370 nm and emission at 445 nm by LS45 fluorescence spectrophotometer (PerkinElmer).

### 2.9. Determination of Oxidative Stress Biomarkers in the Brain

Brain catalase (CAT) activity [36], reduced glutathione (GSH) [37], and malondialdehyde (MDA) [38] content in the brain were determined colorimetrically.

### 2.10. Statistical Analysis

Data are expressed as mean ± SEM. Statistical analysis was performed using one-way analysis of variance (ANOVA) followed by Tukey's post hoc test at *P* < 0.05 using Graphpad Prism software.

## 3. Results

### 3.1. Body Weight

In the current study, diabetes caused a significant decrease in body weight in comparison to control rats (239 versus 302 gm). Meanwhile, treatment with alagebrium, perindopril, and gliclazide did not cause any significant change in body weight compared to diabetic rats as shown in Table 1.

### 3.2. Blood Glucose

Blood glucose level was significantly increased after STZ injection in comparison to control rats (552 versus 123 mg/dL). However, treatment with alagebrium, perindopril, and gliclazide did not cause any significant change in blood glucose level compared to diabetic rats (Table 1).

### 3.3. Blood Pressure

#### 3.3.1. Diastolic Blood Pressure

The present study has demonstrated a significant increase in diastolic blood pressure in diabetic rats compared to control rats (110 versus 77 mmHg). On the other hand, treatment with perindopril caused a significant decrease in diastolic blood pressure compared to diabetic rats (81 versus 110 mmHg) as shown in Table 1.

#### 3.3.2. Systolic Blood Pressure

In addition, diabetes caused a significant increase in systolic blood pressure in comparison to control rats (126 versus 105 mmHg), while treatment with alagebrium, perindopril, and gliclazide did not cause any significant change in systolic blood pressure compared to diabetic rats as shown in Table 1.

### 3.4. Serum Advanced Glycation End Products (AGEs)

Diabetes was associated with a significant increase in serum AGEs level in comparison to control rats (128 versus 47 units). Meanwhile, treatment with alagebrium, perindopril, and gliclazide caused a significant decrease in serum AGEs level compared to diabetic rats (103, 106, and 121 versus 128 units, resp.) as shown in Table 1.

### 3.5. Brain Advanced Glycation End Products (AGEs)

**Figure 1:**
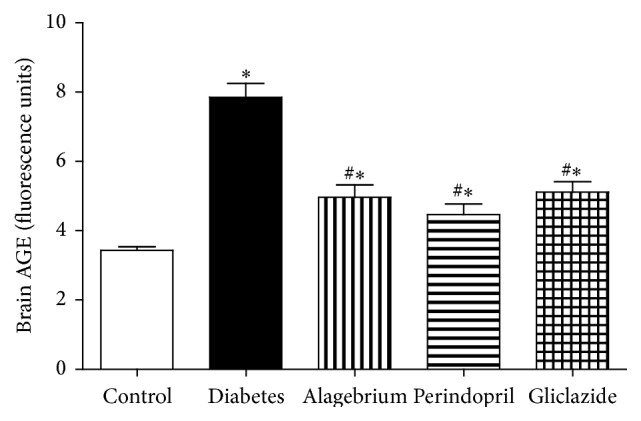
Effects of alagebrium (10 mg/kg), perindopril (4 mg/kg), or gliclazide (10 mg/kg) on brain AGEs level in STZ-induced diabetes in rats. Data are presented as mean ± SEM (*n* = 6). ^*∗*^Significantly different from control group. ^#^Significantly different from diabetic group at *P* < 0.05 using ANOVA followed by Tukey's post hoc test.

The elevation in serum AGEs was also associated with an increase in brain AGEs level in diabetic rats in comparison to control rats (7.8 versus 3.4 units). Similarly, treatment with alagebrium, perindopril, and gliclazide caused a significant decrease in serum AGEs level compared to diabetic rats (5, 4.5, and 5.1 versus 7.8 units, resp.) as shown in Figure 1.

### 3.6. Neuronal Degeneration

**Figure 2:**
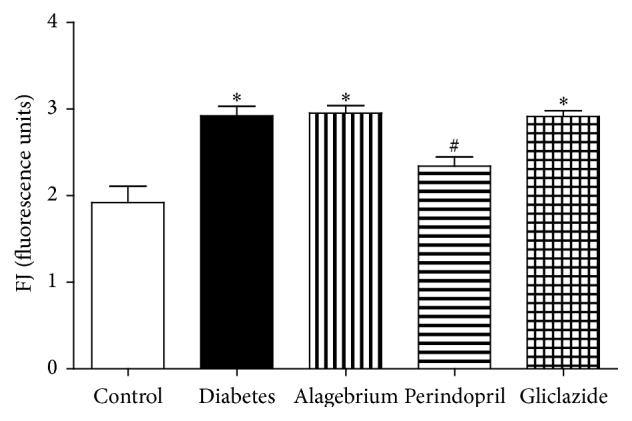
Effects of alagebrium (10 mg/kg), perindopril (4 mg/kg), or gliclazide (10 mg/kg) on neuronal degeneration (Fluoro-Jade fluorescence) in STZ-induced diabetes in rats. Data are presented as mean ± SEM (*n* = 6). ^*∗*^Significantly different from control group. ^#^Significantly different from diabetic group at *P* < 0.05 using ANOVA followed by Tukey's post hoc test.

In the current study, diabetes caused a significant increase in neuronal degeneration as evidenced by the increase in Fluoro-Jade (FJ) fluorescence in comparison to control rats (2.9 versus 1.9 units). Meanwhile, only treatment with perindopril caused a significant decrease in FJ fluorescence as compared to diabetic rats (2.3 versus 2.9 units) as shown in Figure 2.

### 3.7. Behavioural Change in Y-Maze

**Figure 3:**
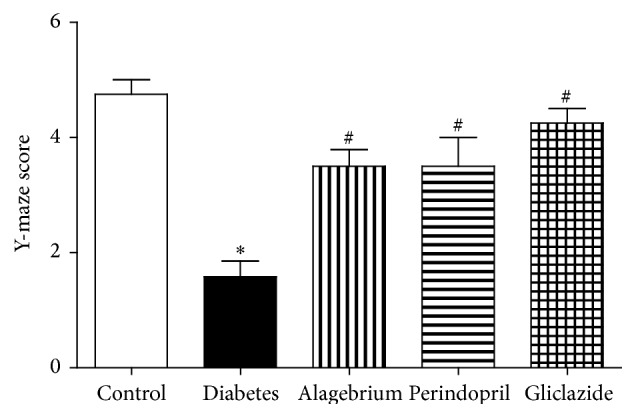
Effects of alagebrium (10 mg/kg), perindopril (4 mg/kg), or gliclazide (10 mg/kg) on Y-maze score in STZ-induced diabetes in rats. Data are presented as mean ± SEM (*n* = 6). ^*∗*^Significantly different from control group. ^#^Significantly different from diabetic group at *P* < 0.05 using ANOVA followed by Tukey's post hoc test.

The present study has shown that diabetes caused a significant decrease in Y-maze score in comparison to control rats (1.6 versus 4.7 units). On the other hand, treatment with alagebrium, perindopril, and gliclazide caused a significant increase in Y-maze score compared to diabetic rats (3.5, 3.5, and 4.2 versus 1.6, resp.) as shown in Figure 3.

### 3.8. Brain Oxidative Stress

#### 3.8.1. Catalase

**Figure 4:**
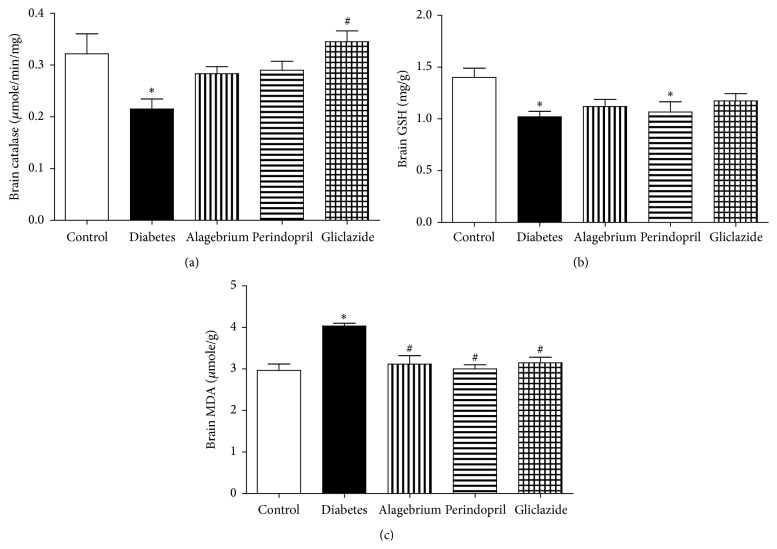
Effects of alagebrium (10 mg/kg), perindopril (4 mg/kg), or gliclazide (10 mg/kg) on (a) brain catalase activity, (b) brain GSH content, and (c) brain MDA content in STZ-induced diabetes in rats. Data are presented as mean ± SEM (*n* = 6). ^*∗*^Significantly different from control group. ^#^Significantly different from diabetic group at *P* < 0.05 using ANOVA followed by Tukey's post hoc test.

Administration of STZ caused a significant decrease in brain catalase activity in comparison to control rats (0.21 versus 0.32 *μ*moles/min/mg). Brain catalase activity was significantly increased following treatment with gliclazide in comparison to diabetic rats (0.34 versus 0.21 *μ*moles/min/mg) as shown in Figure 4(a).

#### 3.8.2. GSH

Similarly, diabetes caused a significant decrease in brain GSH content in comparison to control rats (1 versus 1.4 units). However, treatment with alagebrium, perindopril, and gliclazide did not cause any change in brain GSH content compared to diabetic rats as shown in Figure 4(b).

#### 3.8.3. MDA

In the current study, diabetes caused a significant increase in brain MDA content in comparison to control rats (4 versus 3 *μ*moles/g). In addition, treatment with alagebrium, perindopril, and gliclazide caused a significant decrease in brain MDA content in comparison to diabetic rats (3.1, 3, and 3.1 versus 4 *μ*moles/g, resp.) as shown in Figure 4(c).

## 4. Discussion

The present study was designed to investigate the impact of diabetes on blood pressure and some central nervous system (CNS) functions. Also, this study investigated the possible beneficial effects of alagebrium, a highly potent AGE-cross-link breaker that has the ability to reverse already-formed AGE cross-links [39–42], and perindopril, a brain-penetrating angiotensin-converting enzyme (ACE) inhibitor [43] known to increase plasma sRAGE level [21], against these diabetic complications.

In the current study, diabetes was associated with a decrease in body weight which was not altered by treatment with alagebrium, perindopril, and gliclazide. These findings keep pace with previous studies in diabetic rats [44, 45].

In addition, STZ administration caused a significant increase in blood glucose level as previously described [46–48]. The elevation in blood glucose level following STZ injection was not altered by treatment with alagebrium, perindopril, and gliclazide at the tested doses and timepoint.

The present investigation has shown that diabetes caused a significant increase in diastolic blood pressure, which was decreased by treatment with perindopril only confirming its hypotensive effect [25]. Similarly, diabetes caused a significant increase in systolic blood pressure which was not altered by any of the treatments used. Elevation of blood pressure was previously reported following induction of diabetes [3, 49, 50] and STZ-induced hypertension was prevented by perindopril treatment [3].

AGEs are the end product of a nonenzymatic reaction with sugar derivatives which leads to irreversible protein-protein cross-links [51]. When AGEs link to long-lived proteins, such as collagen in the arterial wall, they contribute to arterial stiffening [52]. Furthermore, AGEs bind to specific AGE-binding receptors on endothelial cells and quench nitric oxide, thereby leading to endothelial dysfunction [25]. The increased formation of advanced glycation end products (AGEs) constitutes a potential mechanism of hyperglycaemia-induced micro- and macrovascular disease in diabetes [53].

Diabetes was associated with an increase in serum AGEs level after STZ administration, which was decreased by treatment with alagebrium and perindopril. Several studies have demonstrated the involvement of AGEs in micro- and macrovascular complications of diabetes [54] and similar elevation in AGEs was previously reported following induction of diabetes [44, 55].

Alagebrium breaks established AGE cross-links between proteins [56]. Alagebrium therapy was previously shown to be associated with reduced AGEs accumulation and RAGE expression in diabetic rats [19]. Previous animal studies and initial phase I and II patient studies demonstrated reduced vascular stiffness and improved endothelial function by alagebrium [57]. Several studies have also shown that ACE inhibition reduced the accumulation of serum AGEs in diabetes, possibly via effects on oxidative pathways [20, 58].

In the current study, diabetes caused a significant increase in brain AGEs which was prevented by treatment with alagebrium, perindopril, and gliclazide.

AGEs were reported to be directly neurotoxic to cultured neurons [59] promoting neuronal cell death and contributing to neurodegenerative disorders such as Alzheimer's disease (AD) [60]. This explains why diabetes caused a significant increase in neuronal degeneration as evidenced by Fluoro-Jade (FJ) fluorescence, which was prevented only by treatment with perindopril.

The ability of perindopril to abolish the enhanced neuronal degeneration might be partly mediated by its ability to reduce AGEs level, increase the production of sRAGE [21], and reduce elevated blood pressure [3] as previously demonstrated.

The changes in neuronal degeneration were accompanied by parallel changes in behavior as diabetes caused a significant decrease in Y-maze score. Similar alterations in memory were previously reported following induction of diabetes [61, 62].

Rats treated with perindopril had near-normal performance in the Y-maze. Perindopril was previously reported to prevent cognitive impairment in AD mouse model through the suppression of microglia/astrocyte activation and the attenuation of oxidative stress [43]. In addition, antihypertensive treatment based on perindopril was demonstrated to reduce cognitive decline in patients with cerebrovascular disease [63].

Surprisingly, alagebrium and gliclazide also improved rat performance in the Y-maze although they had no influence on neuronal degeneration measured by Fluoro-Jade fluorescence. Alagebrium was shown to ameliorate A*β*-induced neuronal death in rat brains [60] which is linked to Alzheimer's disease and associated memory defects [64]. However, in the present study, alagebrium had no effect on neuronal degeneration which indicates that this effect might be attributed to another action and needs further investigation. Gliclazide was shown to stimulate peroxisome proliferator activated receptor gamma (PPAR-*γ*) and exert antiamyloidogenic and anti-inflammatory effects, which may play a role in delaying and reducing the risk of neurodegeneration as previously demonstrated [65]. This could explain the effectiveness of gliclazide in improving rat performance in the Y-maze without alteration in neuronal degeneration.

This research has shown that diabetes caused a state of brain oxidative stress as evidenced by the decrease in brain catalase activity, decrease in brain GSH content, and increase in brain MDA content. Treatment with gliclazide caused a significant increase in brain catalase activity and a decrease in brain MDA activity, while treatment with alagebrium and perindopril caused a decrease in brain MDA activity suggesting antioxidant action.

STZ is known to increase production of ROS [66] and MDA [3], while reducing antioxidant capacity [67] as GSH level [3] in diabetic animals.

In addition to the ligation of RAGE, AGEs may increase generation of ROS by decreasing activities of superoxide dismutase (SOD) and catalase and diminishing glutathione stores [68].

Diabetes-associated increase in superoxide production was prevented by alagebrium [19] which also increased the availability of GSH [40], glutathione peroxidase, and superoxide dismutase activities in aging rats and reduced oxidative stress [41]. This antioxidant action of alagebrium might partly explain its neuroprotective effect that caused improvement in the performance in Y-maze without alteration in neuronal degeneration and further studies are required to explain this phenomenon.

Treatment with perindopril was shown to prevent STZ-induced oxidative stress in rats by increasing GSH and decreasing MDA levels [3]. In addition, it reduced oxidative stress and increased plasma antioxidant capacity in hypertensive patients [69].

Gliclazide has antioxidant effect [70] acting as a general free radical scavenger [71, 72], which was shown to prevent the increase of MDA and SOD during diabetes [73].

Although the results from experimental studies concerning alagebrium [74, 75] were promising, safety and/or efficacy in clinical studies seems to be a concern [53, 76]. Alagebrium showed beneficial effects on a range of cardiovascular variables in hypertensive individuals [56, 57], whereas other clinical trials in heart failure patients did not show any beneficial effects [77, 78]. In addition, the cardioprotective effects of 1-year exercise training in previously sedentary older subjects were not potentiated by alagebrium [51].

## 5. Conclusion

Diabetes is associated with vascular and behavioral complications including hypertension and dementia which might be mediated by brain oxidative stress and neuronal degeneration. Although perindopril was effective in reducing the elevated blood pressure induced by diabetes, this effect could not be fully attributed to amelioration of AGEs signaling since perindopril is a known ACEI and alagebrium was not effective against elevated blood pressure.

Blockade of AGEs signaling by alagebrium and perindopril in rats as late as 8 weeks after diabetes induction was effective in reducing CNS complications only which suggests the possible use of these drugs to manage diabetic central complications together with conventional antidiabetic therapies. Whether similar effects would be observed in human diabetic patients is the main question and needs further investigation.

## Figures and Tables

**Table 1:** Effects of alagebrium (10 mg/kg), perindopril (4 mg/kg), or gliclazide (10 mg/kg) on body weight, blood glucose level, blood pressure (diastolic and systolic), and serum AGEs level in STZ-induced diabetes in rats. Data are presented as mean ± SEM (*n* = 6).

Parameter	Control	Diabetic	Alagebrium	Perindopril	Gliclazide
Body weight (gm)	301.6 ± 9.1	238.9 ± 8.8^*∗*^	241.8 ± 8.6^*∗*^	230.5 ± 7.2^*∗*^	220.5 ± 7.5^*∗*^
Blood glucose level (mg/dL)	122.7 ± 5.3	552.3 ± 27.7^*∗*^	562 ± 19.3^*∗*^	555.8 ± 18.8^*∗*^	487.8 ± 63.5^*∗*^
Diastolic blood pressure (mmHg)	77.5 ± 2.8	110.3 ± 6.4^*∗*^	111.5 ± 7.6^*∗*^	81.4 ± 3.9^#^	93.8 ± 8.5
Systolic blood pressure (mmHg)	105.1 ± 3.1	125.6 ± 4.1^*∗*^	133.8 ± 3.9^*∗*^	108.7 ± 3.4	115 ± 5.3
Serum AGEs (fluorescent units)	46.8 ± 1.7	128.5 ± 5.3^*∗*^	103.5 ± 4.5^*∗*#^	106.2 ± 3.7^*∗*#^	120.8 ± 2.9^*∗*^

^*∗*^Significantly different from control group. ^#^Significantly different from diabetic group at *P* < 0.05 using ANOVA followed by Tukey's post hoc test.
